# The development of an online intellectual humility intervention for religious or spiritual conflict

**DOI:** 10.1016/j.invent.2025.100871

**Published:** 2025-09-09

**Authors:** Arcadia K. Lee, Brandon Wong, Elizabeth J. Krumrei-Mancuso

**Affiliations:** Pepperdine University, United States

**Keywords:** Online intervention, Religious/spiritual struggle, Religious/spiritual conflict, Intellectual humility, Perceived stress, Psychological distress

## Abstract

Interpersonal religious/spiritual (R/S) conflicts are relatively common and have serious mental health implications. Recent theories suggest that intellectual humility might mitigate R/S conflict and ameliorate negative outcomes. This paper delineates the development and implementation of *Grounded to Grow*, an asynchronous online intervention designed to help individuals navigate R/S conflicts and to minimize associated psychological distress. This paper outlines the theoretical framework used to create the intervention, describes the intervention, and highlights the planned methods for examining the effectiveness of the intervention. *Grounded to Grow* is appropriate for religious, spiritual, non-religious, and non-spiritual individuals. This secular, yet spiritually-sensitive approach to addressing R/S conflicts offers inclusive and personalized support, with the potential to enhance well-being across psychological, social, and spiritual domains.

## Highlights

• Interpersonal conflicts about religion/spirituality have known mental health implications.
• Intellectual humility may mitigate such conflicts and their outcomes.
• We describe an online intellectual humility intervention for religious/spiritual conflict.
• Preliminary participant feedback is positive.

## 1 Introduction

Interpersonal religious or spiritual (R/S) conflicts are common. In fact, a recent study of 18,000 U.S. adults indicated almost half (45.5 %) had experienced a R/S conflict in the past few weeks (Pargament and Exline, 2021). Rates are even higher among religious communities, with as many as 89 % of individuals reporting having experienced a R/S conflict (Dollahite et al., 2019). R/S conflict involves struggling in an interpersonal relationship due to closely held R/S beliefs, practices, or behaviors (Pargament, 2007). They can manifest as intellectual disagreements, clashes in beliefs or values, negative experiences with religious people or institutions, or anger toward organized religion (Exline et al., 2014; Wilt et al., 2022).

Of note, R/S conflicts are not limited to individuals who identify as R/S. Atheists also report experiencing such conflicts, often at rates comparable to their religious counterparts (Sedlar et al., 2018). R/S conflicts among atheists may appear as disagreements with individuals who identify as R/S or as a reaction to religious-based prejudice they experience (Exline et al., 2014). Therefore, interventions targeting R/S conflict are relevant to religious and non-religious individuals alike.

### 1.1 The mental health implications of religious/spiritual conflicts

There are numerous benefits to holding R/S beliefs (Ellison and Fan, 2008; Krumrei-Mancuso et al., 2025b; VanderWeele, 2017). At the same time, interpersonal conflict about matters considered to be moral or sacred are associated with a sense of threat (Pargament et al., 2017; Skitka et al., 2021). R/S conflicts can involve perceptions of threat (Pargament, 2007), feelings of sacrilege (Haney and Rollock, 2020), a sense of uncertainty surrounding fundamental beliefs (Bockrath et al., 2022), and social alienation (Exline et al., 2014). Perhaps for these reasons, individuals going through R/S conflict experience increased levels of anxiety, depression, distress, loneliness, negative social interactions, and isolation, as well as decreased life satisfaction and self-esteem (Sedlar et al., 2018; Exline et al., 2014; Wilt et al., 2022; Stauner et al., 2020).

Although the mental health implications of R/S conflicts are documented, there is limited specialized support available. This is likely due to clinicians receiving little training about R/S issues (Oxhandler et al., 2015; Schafer et al., 2011). Indeed, one study of psychotherapists indicated 52 % -81 % received little or no R/S competency training (Vieten et al., 2016). Considering the vast prevalence of R/S conflicts and their negative impact on mental health, there is a need for tailored interventions to help people understand, cope with, and respond to this particular type of conflict.

### 1.2 Intellectual humility as a potential mechanism for reducing religious/spiritual conflicts

One promising, yet unexamined method for mitigating the negative mental health outcomes of R/S conflicts involves cultivating intellectual humility (IH). IH has been conceptualized as an intellectual virtue, disposition, trait, and state. The most common defining characteristic is an awareness of personal intellectual limitations (Porter et al., 2021). IH involves recognizing one's beliefs are fallible and having a level of attentiveness to one's intellectual limitations and their consequences (Haggard et al., 2018; Krumrei-Mancuso and Rouse, 2016; Leary et al., 2017). Given how closely R/S beliefs and values are held, R/S conflict presents a particularly challenging, yet critical context for developing IH (Church, 2018; Dormandy, 2018; Hook et al., 2015). IH is associated with helpful qualities, such as having a more accurate understanding of what one does and doesn't know and experiencing more intrinsic motivation to learn new information (Krumrei-Mancuso et al., 2020). Further, IH is associated with prosocial characteristics and attitudes such as empathy, altruism, and benevolence (Krumrei-Mancuso, 2017). In the contentious context of R/S conflict, this seems to translate into greater forgiveness of conflict partners and even increased attitude change. Specifically, research suggests that IH among those hurt by the transgressions of a religious leader, as well as the perceived IH of a religious leader who engages in a transgression, are associated with forgiveness of the leader's transgressions (Hook et al., 2015). Similarly, the presence of both self-reported IH and the perceived IH of a religious conflict partner has been shown to encourage forgiveness (Zhang et al., 2015) and shifts in attitudes toward a middle ground (Rodriguez et al., 2017). These findings demonstrate the importance of IH in the context of R/S conflicts, particularly when working toward conflict resolution.

### 1.3 Utility and feasibility of an online intellectual humility intervention for R/S conflict

Technological advancements have enabled the field of psychology to provide accessible and effective Internet-based services (Löchner et al., 2025). Online mental health services have several advantages, including 24/7 accessibility, reduced travel time and associated costs, flexibility to participate at one's own pace, the option for anonymity, reduced need for direct involvement from mental health professionals, and greater affordability (Andersson and Titov, 2014; Cuijpers et al., 2009).

In addition to these general benefits, internet interventions are particularly useful when it comes to R/S conflicts causing psychological distress for two reasons. First, there is a lack of specialized R/S competency training among most clinicians (Oxhandler et al., 2015; Schafer et al., 2011; Vieten et al., 2016). Internet interventions allow clinicians who are specialized in this area to create and validate intervention content to the benefit of individuals who are not able to find clinicians in their local area who have training or experience working with R/S concerns. Second, the stigma surrounding R/S conflicts may deter individuals from seeking support in typical settings, such as mental health counseling or support from religious communities (Milner et al., 2019). An internet intervention can offer specialized support in a modality that decreases the stigma-related barriers to seeking help. Such interventions can further work to attenuate the shame and stigma associated with R/S conflicts, which can broaden individuals' opportunities for seeking support through additional means (e.g. through mental health services, religious communities, or support groups).

#### 1.3.1 Existing interventions

A combined search of PsycInfo and Academic Search Complete for empirical articles in peer-reviewed journals with the search: (religion or spirituality) AND (struggle or conflict) AND (intervention), yielded 226 results. Eight of these studies described interventions targeting R/S struggles of various kinds (Ano et al., 2017; Betz et al., 2019; Bowland et al., 2011; Dworsky et al., 2013; Harris et al., 2018; Kim et al., 2025; Murray-Swank and Pargament, 2005; Reist et al., 2019). Of these interventions, only four included a focus on R/S struggles in interpersonal contexts, but did so in addition to focusing on R/S struggles in other domains, such as struggles related to God, morality, or meaning (Betz et al., 2019; Bowland et al., 2011; Dworsky et al., 2013; Kim et al., 2025; Reist et al., 2019^1^). Further, most of these interventions were tailored to R/S struggles in the context of particular life challenges or crises. Betz et al. (2019) targeted spiritual struggles among monotheistic parents of children with cystic fibrosis. This intervention consisted of three telephone conversations with a chaplain, with each conversation addressing intrapersonal, interpersonal, and divine spiritual struggles. Dworsky et al. (2013) developed a multi-modal in-person group intervention for college students experiencing intrapersonal, interpersonal, or divine spiritual struggles. The same intervention was subsequently implemented by Reist et al. (2019) among partially hospitalized adult psychiatric patients. Kim et al. (2025) examined an in-person, peer-led R/S intervention designed for veterans with PTSD, with the goal of minimizing suicidal ideation and suicide attempts that might be associated with a broad range of R/S struggles. Finally, a qualitative study examined an in-person intervention for female survivors of abuse targeting R/S struggles associated with alienation or self-stigma with the goal of increasing positive coping strategies and maintaining connection to religion or spirituality despite struggle (Bowland et al., 2011).

Each of these studies has helped establish the feasibility of interventions addressing R/S struggles. Given that each targeted a fairly unique population, however, there is room to expand this work to the general population and to develop interventions tailored to particular types of R/S struggles people might be encountering at a given time. In addition, it would be of great benefit to provide an option via the internet. *Grounded to Grow* was developed as an internet intervention for individuals with psychological distress resulting from R/S conflict.

To date, there have been no interventions targeting IH as a mechanism in the context of R/S conflict. A combined search for empirical articles in peer-reviewed journals in PsycInfo and Academic Search Complete with the search: (intellectual humility) AND (intervention), yielded 17 results. Among these, only two IH interventions were described. Gómez et al. (2023) described the theoretical development of an online, self-paced intervention designed to facilitate intellectual humility in educational settings. This intervention consists of eight lessons, each centered around a different activity to cultivate IH and lasting between 30 and 60 min. Welker et al. (2023) evaluated this intervention and observed increases in intellectual humility over the course of the intervention in the context of political disagreements. In addition, Mendonça et al. (2023) examined a three-month online intervention for adults consisting of weekly exercises and found that the intervention was able to increase both state and trait IH. It is promising that these early studies have offered evidence of the feasibility and efficacy of internet-based IH interventions. *Grounded to Grow* expands this work further and is novel in that it focuses on how IH can help individuals in the context of R/S conflict by reducing the frequency and intensity of R/S conflict, helping individuals cope with R/S conflict, and minimizing the negative psychological impact of R/S conflict.

### 1.4 Aims

*Grounded to Grow* is a recently developed online intervention accessible via computer and mobile devices. The program is currently under evaluation. In this paper, we delineate the theoretical framework, design, and development of this online intervention for individuals experiencing R/S conflicts. We include preliminary participant feedback and outline plans for examining the intervention's feasibility and efficacy.

## 2 The intervention

### 2.1 Team and development

The *Grounded to Grow* online intervention was developed over eight months during 2023–2024 by a team of psychologists spanning academic and clinical settings. The team consisted of three licensed clinical psychologists (Elizabeth Krumrei-Mancuso, professor of psychology; Carol Ann Caprini, psychologist in private practice; and Maria Gear Haugen, psychologist for Veteran Affairs), one social psychologist (Rosemond Lorona, associate professor of psychology), and two post-bachelor's in research positions (Arcadia Lee and Brandon Wong). This team was assembled to bring together the expertise needed to reach several goals for the design of this online intervention, including presenting material clearly and in a manner conducive to learning, helping participants process heightened emotions related to the R/S that might detract from their ability to focus on the intervention content, offering psychoeducation about R/S conflict, and providing effective education and intervention strategies for increasing IH.

### 2.2 Ethical and cultural considerations

The intervention is not religious or spiritual in nature, but is respectful of participants' religion or spirituality. The language and content of the intervention was designed to be appropriate for individuals of any or no R/S affiliation. The intervention is based on an understanding of religion and spirituality as ways in which people engage with that which they hold sacred (Pargament, 1997) and that there is great diversity in what people hold sacred (Krumrei-Mancuso et al., 2025c). This approach allowed us to be inclusive of many R/S identities, affiliations, and beliefs of participants, which was key to ensuring the cultural sensitivity of the intervention. In addition, to increase accessibility and comprehension, each component of the intervention involving speech is equipped with closed captions.

Given the deeply personal and often sensitive nature of R/S conflicts, procedures are in place to ensure participants are supported throughout the intervention. Prior to starting the intervention, all participants are provided with national and local resources related to mental health, domestic violence, and emotional abuse. As participants progress through the intervention, their qualitative responses are monitored for indicators of emotional or psychological distress. Individuals whose responses are flagged receive individualized follow-up from the team.

### 2.3 Structure and design

The *Grounded to Grow* intervention consists of four asynchronous modules delivered on a virtual platform. Activities are embedded within these weekly modules and three midweek activities. The modules are designed to be completed within one hour and can be re-accessed if needed. At the end of each module, participants are provided with additional resources for optional further reading and exploration. At the mid-point between weekly modules, participants receive an email with mid-week activities. These activities are used to reinforce module content and provide opportunities for practice that take 20–30 min to complete.

Each module follows a similar structure. The content includes overviews of the module theme and goals, experiential activities, psychoeducation (often through stories and examples), reflective activities, skills practice, integration, conclusions, summaries, overviews of upcoming activities, and optional bibliotherapy.

The intervention uses a multimedia approach. The content is highly interactive and presented through videos, images, graphics, guided auditory experiences, short texts, activities, and guided journaling. Employing multiple modes of delivery has been shown to promote heightened cognition, engagement, and learning (Moreno and Mayer, 1999).

### 2.4 Metaphor of growing a strong tree

The use of metaphors has been employed in various psychotherapies to increase understanding (Stott et al., 2010). *Grounded to Grow* uses the metaphor of a tree and suggests that experiencing a R/S conflict may feel like being a tree caught in a storm. The program addresses various aspects of being a strong tree, including having deep roots, representative of living by one's values; having branches that extend out into the world, which represent one's beliefs; having a thick treetop, which represents one's metacognition; and having a strong, yet flexible trunk which represents wisdom. Overall, the theme of growth may lessen participants' sense of threat associated with noticing and acknowledging their intellectual limitations (Porter and Schumann, 2017).

### 2.5 Intervention content and underlying theory

Though limited, there is some empirical support for mechanisms that might promote IH. *Grounded to Grow* is based on this body of research.

#### 2.5.1 Orientation

A video introduction acquaints participants with the theme, goals, and format of the intervention and introduces the intervention team. The orientation includes a one-minute awe-inducing video that is subsequently played at the beginning of each module. Self-transcendent experiences such as wonder, elevation, and awe have not only been shown to facilitate IH (Kim et al., 2022; Krumrei-Mancuso et al., 2023) but also to increase people's desire to become more intellectually humble (Kim et al., 2022). As self-transcendence tends to be elicited by vast, complex, and majestic nature scenes, the video showcases lightning, mountainous landscapes, changing seasons, and waterfalls. The video images are arranged to *Victory*, composed by Thomas Bergesen, which has also been shown to elicit a sense of awe (Ji et al., 2019).

Following the orientation video, participants schedule a one-on-one video call with a team member. The goals of this meeting are to build rapport with the participant, make sure participants understand the goals and procedures of the intervention, and answer participant questions. Following the one-on-one meeting, participants in the intervention group receive an email with the link to the first module. Completing a module generates two automated emails, one with the link to the midweek activity (3 days later) and one with the link to the subsequent module (7 days later) until the last module is complete.

#### 2.5.2 Module 1: values as tree roots

The goal of the first module is to create a welcoming space where participants feel comfortable exploring their R/S conflict. Efforts to promote IH can backfire if they pose an existential threat to individuals (e.g., see Ballantyne, 2023), particularly when it comes to core beliefs and values (Krumrei-Mancuso and Worthington Jr., 2023). In contrast, meaning affirmations are associated with reduced defensiveness and greater openness (Van Tongeren et al., 2014). Perhaps for this reason, research suggests that affirming people's values can increase IH (Hanel et al., 2023; Marie et al., 2022). Value-affirmations have also been shown to facilitate coping with perceived threats by promoting self-transcendence and high-level mental construals (Burson et al., 2012; Schmeichel and Vohs, 2009).

Consistent with the module theme that a person's values are like roots that provide a solid foundation and nourishment, this module is designed to help participants identify and live more in line with their core values. Value identification activities are based on the Schwartz values survey, acceptance and commitment therapy inventories, and guided reflection. Next, the module takes participants through several exercises to affirm their most cherished values. These include developing positive affirmations centered on values and finding practical ways to incorporate these affirmations into daily living. To further affirm the participants' values through the mind-body connection, we introduce an adaptation of the 3-S Stretch from Spiritual Self-Schema Therapy (Avants and Margolin, 2004). We ask participants to complete the activity with their self-selected value in mind. With paired body movements, participants are encouraged to focus on the thoughts, words, emotions, actions, and perceptions reflective of their selected value and their openness to receive this value. After a video demonstration and practice with the 3-S Stretch, this activity is incorporated as a midweek activity for the remainder of the intervention.

Module 1 also includes psychoeducation that helps participants understand how clashes in values can contribute to R/S conflicts and how this can elicit a sense of threat (Pargament et al., 2017; Skitka et al., 2021). We also offer tools participants can use to cope with this distress, in order to make space for participants to engage in metacognition about their beliefs and values. Finally, the module focuses on how participants can navigate clashes in values in ways that are true to their core values.

Reinforcing the content of Module 1, the first midweek activity invites participants to engage in a values-based action that they committed to during an exercise in Module 1 and to reflect on the outcome. They are also encouraged to practice their personalized 3-S values-affirming stretch and make use of the value affirmation statements they developed during the module.

#### 2.5.3 Module 2: beliefs as tree branches

The second module uses the motif of tree branches to represent beliefs. We explain that participants can stretch their beliefs out into the world for good, but that they might bump into the branches of others' beliefs, resulting in R/S conflicts. Similar to the approach with values in Module 1, participants explore their personal beliefs and identify some core beliefs they hold about themselves, others, and the world. The module then guides them through a reflection on the core beliefs that feel opposed, threatened, or attacked by the R/S conflict.

This module continues to acknowledge that R/S conflicts can be emotionally straining. The module includes emotion identification, self-compassion, and cognitive detachment exercises. Participants are asked to explore whether the parties involved in the conflict may share some of the same core values, but disagree about how to achieve or demonstrate those values. Similar activities have been employed in various forms of therapy, including cognitive behavioral, mindfulness-based, and acceptance and commitment therapy as a tool to help individuals take a new perspective on the thoughts they hold about a situation (Saulsman, 2025).

We then provide psychoeducation on the concept of IH, exploring the definition and characteristic features in lay terms. We offer a variety of examples of IH from real-world figures (Viktor Frankl, Gandhi, Mother Teresa), given that modeling by trusted figures has been shown to aid in the development of IH (Hall, 2021). The goal is to inspire and motivate participants by observing contexts in which IH is a desirable trait. For example, a clip of a conversation between Desmond Tutu, a Christian, and the Dalai Lama, a Buddhist, is presented. In this video, the two men demonstrate IH as they discuss their differences.

Additionally, Module 2 includes guided visualizations to enhance psychological distancing from the R/S conflict, which has been shown to increase IH (Grossmann et al., 2021). Participants are guided through a process of experiencing the R/S conflict from multiple perspectives: their own, the other person(s), and that of an impartial observer. To further promote psychological distance, participants engage in a journaling exercise in which they write about the conflict from a third-person perspective (referring to themselves as “she” or “he” or by name, etc.). This has been shown to increase metacognitive insights and IH in interpersonal contexts (Grossmann et al., 2021; Lockhart et al., 2016). This journaling activity is then incorporated as a recurring mid-week activity, given that self-distanced journaling is more effective when completed habitually over multiple weeks (Grossmann et al., 2021).

For the second mid-week activity, participants are asked to write a journal entry about their R/S conflict from a distanced, third-person perspective. They are also guided through a self-compassion visualization and a prompt to practice their personalized 3-S values-affirming stretch from Module 1.

#### 2.5.4 Module 3: metacognition as the treetop

The motif of the third module is the treetop, the highest part of the tree, which represents people's potential to reflect on their own beliefs. IH is often thought of as a form of metacognition, in that it consists of people's ability to recognize that their thinking might be wrong or incomplete (Deffler et al., 2016; Leary et al., 2017). Thus, participants are introduced to the idea of considering not only *what* they think (the content of their beliefs), but also *how* they think about their beliefs relevant to their R/S conflict.

Module 3 incorporates psychoeducation about IH with a TED Talk by journalist Kathryn Schulz. This video highlights the errors that all people are prone to in their thinking. We use this video to normalize cognitive limitations and errors in both oneself and one's conflict partner(s). The video is a launching point for exercises in which participants explore how they feel when they realize they've made a cognitive error, what they do to cope with associated discomfort, and how to use their wrongness as an avenue for discovering wonder and awe. Additional exercises explore ways in which IH can help participants minimize the villainization of conflict partners and be more intellectually generous toward themselves and others.

To continue to motivate greater IH, Module 3 invites participants to reflect on a personal IH hero who embodies the characteristics of IH. This exercise helps participants learn to recognize IH, understand the specific qualities that make up IH, and serves as a point of inspiration as they notice how it feels to be around their IH hero. A similar activity was identified by participants of a general humility intervention as enjoyable (Cuthbert et al., 2018).

Midweek Activity 3 includes a guided journal about an instance in which participants discovered they had been wrong in their thinking. They explore the associated emotions and the underlying background experiences and sociocultural factors that may have contributed to these feelings. This activity prompts participants to explore whether and how IH relates to their self-image. In addition, participants continue to journal about their R/S conflict from a distanced, third-person perspective (introduced in Module 2) and engage in their personalized 3-S values-affirming stretch (introduced in Module 1).

#### 2.5.5 Module 4: wise self as the tree trunk

Module 4 uses the motif of a tree trunk to represent the whole self—the strong, yet flexible part of a tree that can sway rather than break in the storm of the R/S conflict. This module focuses on review, integration, and closure. Guided imagery is used to invite participants to imagine crossing paths with a much older and wiser version of themselves. This version of themselves is deeply grounded, aligned with their values, and unafraid to acknowledge and take responsibility for their intellectual mistakes. Adopting a future-oriented perspective is associated with increased IH (Huynh et al., 2016) and has a positive impact on the brain's reward system (Cascio et al., 2016). This future-oriented activity centered on individual growth can help participants think about an openness to discovering intellectual limitations over the lifespan.

Additionally, this module circles back to value-affirming mantras. Participants are encouraged to reflect on their personalized values-mantras and express them in a way that feels meaningful to them, such as portraying the mantra as a drawing, writing it down and decorating it, or moving their body in a way that embodies the mantra.

Module 4 also incorporates a focus on gratitude, which has been shown to cultivate IH through processes of self-transcendence and broadened perspective (Krumrei-Mancuso et al., 2023). Although we are cautious not to imply that the end of the intervention signifies an end to the R/S conflict or associated distress, we invite participants to reflect on whether they have experienced personal growth as a result of their R/S conflict. This module closes with the personalized 3-S values-affirming stretch and invites participants to carry forward the practices and tools from the intervention they found most helpful.

### 2.6 Personalization aspects

Previous studies have suggested that personalization increases participant engagement and positive perceptions of a program (Saleem et al., 2021). Additionally, simple, personalized outreach efforts with details about tasks are known to be effective in demonstrating the researcher's commitment to the participant, thereby aiding in the development of rapport (Ochieng et al., 2021).

Our personalization efforts include one-on-one welcome meetings (see Section 2.5.1), emails including relevant images with motivational texts addressed to participants by name, and intervention activities in which participants are asked to engage based on their specific R/S conflict. For many activities, piped text is used to tailor the experience to participants' context by presenting to participants details they previously provided about their R/S conflict, personal goals, or values.

In addition, during the registration process, participants are presented with the option to receive individualized feedback upon completion of the intervention. This option is offered in recognition that some individuals feel encouraged when they know someone will read their responses, whereas other participants feel more comfortable opening up if their responses remain private. If endorsed, a team member reviews the participants' written responses throughout the modules and emails the participant feedback about ways in which the participant has expressed and grown in IH and places for further reflection. The concept of participant feedback has been employed in previous humility interventions (Cuthbert et al., 2018). In addition, providing participants observer feedback on IH based on their writings may supplement their personal understanding of their IH (Meagher et al., 2015, Meagher et al., 2020).

## 3 Evaluation

*Grounded to Grow* is a novel online intervention that is currently being examined through a preregistered randomized controlled trial at https://osf.io/jqub9. In this section, we describe the procedures being used to evaluate the effectiveness of the intervention.

### 3.1 Evaluation ethics

All research procedures are being performed in compliance with relevant laws and institutional guidelines and were approved by Pepperdine University's Institutional Review Board (Protocol #24-01-2339). Prior to participating in the study, individuals undergo an informed consent process in which they are provided with written explanations followed by a one-on-one video call with a team member to review information about the study and address any questions or concerns.

To ensure confidentiality, each participant is assigned a unique, randomized 7-digit identification number. This allows participants' data to be analyzed without linking participants' responses to their identities within the data files. All data files and the participant directory are stored in encrypted files on password-protected drives and computers.

Given that R/S conflict can be sensitive in nature, we monitor participants' responses on a measure of distress (the Brief Symptom Inventory-18, Derogatis, 2000). We send individualized follow-up with mental health resources to participants who score high on distress (≥ 63 on the Global Severity Index) or who endorse an item on suicidality.

### 3.2 Examination of intervention outcomes

**Fig. 1:**
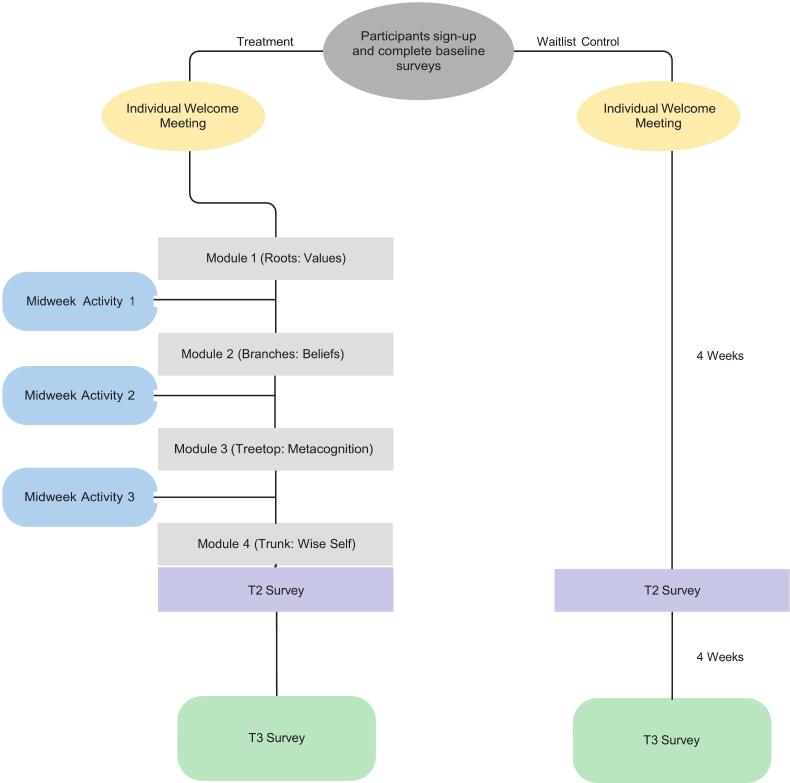
Flowchart of participation in the intervention.

Eligibility criteria for participating include being an adult who is experiencing a R/S conflict that is causing distress and residing within our recruitment region. Eligible participants are randomly assigned to either the intervention or a waitlist control. Fig. 1 depicts the flow through each study arm. Data are collected at three points in time: (1) baseline during intervention registration, (2) immediately after the intervention, and (3) one month after completing the intervention. The waitlist-control participants complete surveys in the same time intervals.

Outcomes are assessed with validated instruments. We will examine levels of intellectual humility in the context of ISS with two scales presented in counterbalanced order: the Intellectual Humility Scale (Leary et al., 2017; e.g., “I question my own opinions, positions, and viewpoints because they could be wrong”) and the Lack of Intellectual Overconfidence subscale of the Comprehensive Intellectual Humility Scale (Krumrei-Mancuso and Rouse, 2016; e.g., “When I am really confident in a belief, there is very little chance that belief is wrong”, reverse coded).

We will examine movement toward resolution of the R/S conflict with a single-item indicator used in previous research (Wilt et al., 2022). Specifically: “Do you think that your interpersonal R/S conflict has been resolved?” with the following response options: “No, it is getting worse,” “No, it is staying about the same,” “Yes, it is partly resolved,” “Yes, it is mostly resolved,” and “Yes, it is totally resolved.”

We will examine self-stigma about R/S conflict the Self-Stigma Scale–Short Form (Mak and Cheung, 2010), which captures stigma across three domains: cognitive, affective, and behavioral (e.g., “My identity as someone who experiences religious/spiritual conflict is a burden to me”).

We will examine stress with the Perceived Stress Scale-4 (Cohen et al., 1983; e.g., “In the past week, how often have you felt that you were unable to control the important things in your life?”).

We will examine psychological distress with the Brief Symptom Inventory-18 (Derogatis, 2000), which includes items in the domains of somatization, depression, and anxiety. We will focus on the global severity score, including all three domains.

We will examine personal growth with a short form of the Posttraumatic Growth Inventory (Cann et al., 2010). Items assess whether the ISS relates to participants' sense of new possibilities, personal strength, appreciation of life, relating to others, and spiritual change.

We will examine participants' level of decision to forgive the conflict partner(s) with the Decision to Forgive Scale (Davis et al., 2015). A sample item is: “I have decided to forgive him/her/them.”

For the current research, we also developed a single face-valid item assessing the normalization of ISS: “How common do you think it is for people to experience religious/spiritual conflict?” With the response options ranging from not very common at all to extremely common. Higher scores indicate participants view R/S conflict as more normal.

In addition to comparing the intervention and control groups on these outcomes, we will examine whether the effectiveness of the intervention differs based on the nature of the content of the R/S conflict, the degree of relational damage done by the R/S conflict, the degree of trust participants have in the conflict partner(s), and participants' levels of religious conservatism.

To test the intervention effectiveness, we will use linear mixed effects models containing time, group assignment, and a time by group interaction. To examine heterogeneity of treatment effects we will examine moderators of the treatment effect with three-way interactions (time by treatment group by moderator).

### 3.3 Recruitment and retention challenges and strategies

Recruitment efforts have included: (1) contacting churches and R/S organizations, (2) advertising on social media (Instagram and Facebook), (3) encouraging participants to share information about the program by word of mouth, and (4) making the program available to students via a research participation system at the affiliated university.

Outreach materials include a link or QR code that leads participants to a website where they can register for the study. Once enrolled, participants can access the intervention through personalized links sent to their email. The intervention is provided free of charge and participants in both arms of the study are offered a $50 gift card for participating in the surveys used to evaluate the intervention.

Although online recruitment can yield larger and more diverse samples (Pollet and Saxton, 2019; Upadhyay and Lipkovich, 2020), there are risks associated with this approach. Previous studies have noted the increasing threat of fraudulent responses as participants may lie to fit the inclusion criteria, participate more than once under aliases, or provide nonsensical responses in an attempt to obtain incentives (Martino et al., 2024; Levi et al., 2021). We quickly observed that we needed to be diligent about detecting and removing ineligible participants to preserve the quality of our data. We have found that targeted recruitment to relevant organizations and social media groups is more likely to reach participants who meet inclusion criteria than general social media advertising, which yields a larger percentage of individuals who seem to sign up only for the incentive.

We are using methods such as those suggested by Teitcher et al. (2015) to identify fraudulent respondents. We first screen registration data for IP addresses and phone numbers in the recruitment regions. Previous researchers have noted that scammers often use Google Voice or disconnected phone numbers to suggest they reside within an approved region (Murdoch-Gibson, 2022). Subsequently, participants engage in a one-on-one video meeting with a team member. This was designed to build rapport and minimize attrition, but has also been very useful for discouraging and detecting participants who attempt to participate in the intervention more than once under different aliases or live outside our eligibility regions. Finally, participants are excluded from the study if they provide problematic written responses on the baseline survey. This includes responses that are nonsensical, identical to other participants' responses, or contain clearly inconsistent or contradictory answers.

### 3.4 Research participants and initial qualitative feedback

Our baseline sample consists of 369 participants, mostly residing in the United States (97.5 %). To date, we have gathered qualitative feedback from 194 participants who completed the full intervention (see Table 1 for demographic information).

**Table 1:** Participant characteristics.

Demographic variable	Full baseline sample (*N* = 369)	Participants providing qualitative feedback (*n* = 194)
**Age**		
Age range	18–53	18–53
Mean age (*SD*)	30.34 (6.19)	30.32 (6.47)
**Gender**		
Male	66.4 %	63.4 %
Female	32.2 %	32.0 %
Non-binary	0.5 %	0.5 %
Prefer not to answer	0.8 %	1.0 %
**Religion**		
Christian	83.8 %	79.4 %
Muslim	1.9 %	2.6 %
Jewish	0.3 %	0.5 %
Buddhist	0.3 %	0.0 %
Agnostic	0.3 %	0.5 %
Other	0.3 %	0.5 %
Prefer not to answer	12.5 %	15.5 %
**Race and ethnicity**		
Black or African American	66.4 %	64.4 %
White	30.9 %	29.4 %
Asian or Asian American	3.5 %	3.7 %
American Indian/Native American or Alaska Native	3.8 %	4.6 %
Native Hawaiian or other Pacific Islander	0.8 %	1.0 %
Other race	0.8 %	0.5 %
Spanish, Hispanic, or Latino origin	8.4 %	8.2 %
Prefer not to answer	0.8 %	0.5 %

Note. Participants were able to select multiple racial categories.

We conducted an initial thematic analysis of responses given to two optional open-ended questions at the end of the final module (see Table 2). The first question is, “Did you learn anything new about yourself through this process?” A predominant theme in the responses included the development of new skills (84 % of responses). Commonly named skills included resilience and empathy, which have both been shown to be positively impacted by higher levels of IH (Lehmann et al., 2025). In addition, 14 % of respondents spontaneously indicated they learned about IH and were able to grow in IH over the course of the intervention. A notable theme in the responses about IH is that participants are expressing greater self-acceptance in the face of their intellectual fallibility. Although this may initially seem in tension with theories of intellectual humility that emphasize the importance of having an appropriate level of concern about one's intellectual fallibility (e.g., Haggard et al., 2018), future research might examine whether acceptance of one's fallibility is a viable path toward taking responsibility for one's intellectual limitations.

**Table 2:** Participant reflections.

Theme	% of responses involving theme	Direct quotes from participants
*Prompt: Did you learn anything new about yourself through this process?*		
Skill development	84 %	• Yes, I learned that I can be more patient and open-minded than I initially thought. I was surprised to find how much I can grow from handling conflicts with empathy and respect. • I learned that I can slow down for a few minutes a day and breathe. It slows down my anxious brain and allows for new ideas and more openness and relaxation. • I learned how to listen to other ideas and consider their beliefs • I learnt that I could be more patient, I used to think I have patience but I come to discover I can still be better • I learnt that putting my positive affirmations into practice really helps in my daily activity.
Intellectual humility	14 %	• I was able to embrace intellectual humility. • I should not be ashamed or embarrassed of “I don't know.” • I observed how important being intellectually humble is and also it gave me a chance to know being wrong is not always wrong. • I learnt that it [intellectual humility] is the ability to realize that I might be wrong. And that every single person has an incomplete picture of reality and if we can embrace that, we rediscover wonder about everything that surrounds us. • I learnt that being wrong is not the end of the world, I have increased my intellectual humility in the past few weeks as I've often practiced our lessons.

*Prompt: Would you like to anonymously share any aspect of your story, journey, insights, or words of encouragement with future workshop participants?*		
Opportunity for personal growth	32 %	• Embrace your conflicts as opportunities for growth. Each challenge can teach you something valuable about yourself and others. Stay open and curious. • There's a lot to learn from this workshop, permit yourself to grow and let go of every grudges. • I encourage you to embrace every step, even the most uncomfortable ones. Spiritual conflict, while painful, can serve as a powerful catalyst for personal growth and self-awareness. Remember that moments of struggle often hold the greatest lessons. • Remember, your spiritual conflict is an opportunity for growth, not a sign of failure. Be gentle with yourself, and trust the journey. You are stronger than you think, and your resilience will surprise you.
Learn something new	29 %	• The journey was a really valuable one in terms of learning. It transformed the way I used to observe, think and learn.... It has indeed helped me greatly in my profession and personal life as well. • Be open to learn and to be corrected. Do not shy away from the truth. • Yes, would like them to take this workshop so serious because it's a life changer. Anyone that doesn't know how to relate with people positively will learn after this workshop. • The workshop will help them know how to control emotions and avoid conflict.

Note. The intervention was labeled an online workshop for participants.

The second qualitative question we examined is, “Would you like to anonymously share any aspect of your story, journey, insights, or words of encouragement with future workshop participants?” A notable theme expressed by 32 % of respondents was that conflicts can be an opportunity for personal growth and development, suggesting a growth mindset among participants. There is limited evidence to suggest this might relate to greater IH (Porter and Schumann, 2017). A second theme we observed was that participants answered this question by describing things they had learned (29 %). This echoed themes from the first qualitative question and emphasizes the importance participants placed on gaining new insights and skills through the intervention.

## 4 Discussion

The goal of this paper was to delineate the development of *Grounded to Grow*, an online intervention for cultivating IH in order to decrease the prevalence and intensity of R/S conflicts and associated psychological distress. The intervention was developed by a team of researchers and clinicians on the basis of indications that IH is relevant to resolving R/S conflicts (Hook et al., 2015; Rodriguez et al., 2017; Zhang et al., 2015).

Although we have noted the many advantages of online interventions when it comes to accessibility, flexibility, affordability, and anonymity (Andersson and Titov, 2014; Cuijpers et al., 2009), there are also limitations imposed by the need for participants to have access to a technological device, data/Wi-Fi, and be comfortable using technological devices in order to participate. In addition, the intervention was designed by a team from the U.S. with limited gender and racial diversity. Despite these limitations, the *Grounded to Grow* intervention has several strengths. Content based on specialized clinical training and empirical evidence, multimodal delivery, personalization, remote access, and the intervention being relatively short are qualities we hope will contribute to beneficial outcomes.

Initial qualitative feedback from participants is promising, but future work will determine the effectiveness of the intervention. If effective, *Grounded to Grow* will expand internet-based services in a domain for which little specialized support is available, namely IH in the context of R/S conflict. As research in this domain advances, future work might focus on creating similar interventions targeting collective-level IH (e.g., Krumrei-Mancuso et al., 2025a), which might be particularly useful to communities and organizations within religiously pluralistic societies.

## Declaration of competing interest

The authors declare that they have no known competing financial interests or personal relationships that could have appeared to influence the work reported in this paper.

## Footnotes

1 Note. Dworsky et al. (2013) and Reist et al. (2019) examined the same intervention among different populations.

## References

[bb0005] Andersson G., Titov N. (2014). Advantages and limitations of Internet-based interventions for common mental disorders. World Psychiatry.

[bb0010] Ano G.G., Pargament K.I., Wong S., Pomerleau J. (2017). From vice to virtue: evaluating a manualized intervention for moral spiritual struggles. Spiritual. Clin. Pract..

[bb0015] Avants S.K., Margolin A. (2004). Development of spiritual self-schema (3-S) therapy for the treatment of addictive and HIV risk behavior: a convergence of cognitive and buddhist psychology. J. Psychother. Integr..

[bb0020] Ballantyne N. (2023). Recent work on intellectual humility: a philosopher’s perspective. J. Posit. Psychol..

[bb0025] Betz J., Szczesniak R., Lewis K., Pestian T., Bennethum A.S., McBride J., Grossoehme D.H. (2019). Feasibility and acceptability of a telephone-based chaplaincy intervention to decrease parental spiritual struggle. J. Relig. Health.

[bb0030] Bockrath M.F., Pargament K.I., Wong S., Harriott V.A., Pomerleau J.M., Homolka S.J., Chaudhary Z.B., Exline J.J. (2022). Religious and spiritual struggles and their links to psychological adjustment: a meta-analysis of longitudinal studies. Psychol. Relig. Spiritual..

[bb0035] Bowland S., Biswas B., Kyriakakis S., Edmond T. (2011). Transcending the negative: Spiritual struggles and resilience in older female trauma survivors. J. Relig. Spiritual. Aging.

[bb0040] Burson A., Crocker J., Mischkowski D. (2012). Two types of value-affirmation: implications for self-control following social exclusion. Soc. Psychol. Personal. Sci..

[bb0045] Cann A., Calhoun L.G., Tedeschi R.G., Taku K., Vishnevsky T., Triplett K.N., Danhauer S.C. (2010). A short form of the Posttraumatic Growth Inventory. Anxiety Stress Coping.

[bb0050] Cascio C.N., O’Donnell M.B., Tinney F.J., Lieberman M.D., Taylor S.E., Strecher V.J., Falk E.B. (2016). Self-affirmation activates brain systems associated with self-related processing and reward and is reinforced by future orientation. Soc. Cogn. Affect. Neurosci..

[bb0055] Church I.M. (2018). Intellectual humility and religious belief. J. Psychol. Theol..

[bb0060] Cohen S., Kamarck T., Mermelstein R. (1983).

[bb0065] Cuijpers P., Marks I.M., van Straten A., Cavanagh K., Gega L., Andersson G. (2009). Computer-aided psychotherapy for anxiety disorders: a meta-analytic review. Cogn. Behav. Ther..

[bb0070] Cuthbert A.D., Davis E.B., Aten J.D., Short A., Yarborough C.A., Lavelock C.R., Worthington E.L., Davis D.E., Hook J.N., Van Tongeren D.R. (2018). Cultivating humility in religious leaders: the effectiveness of a spiritually integrated positive psychology intervention. Spirit. Clin. Pract..

[bb0075] Davis D.E., Hook J.N., Van Tongeren D.R., DeBlaere C., Rice K.G., Worthington E.L. (2015). Making a decision to forgive. J. Couns. Psychol..

[bb0080] Deffler S.A., Leary M.R., Hoyle R.H. (2016). Knowing what you know: intellectual humility and judgments of recognition memory. Personal. Individ. Differ..

[bb0085] Derogatis L.R. (2000).

[bb0090] Dollahite D.C., Marks L.D., Young K.P. (2019). Relational struggles and experiential immediacy in religious American families. Psychol. Relig. Spiritual..

[bb0095] Dormandy K. (2018). Does epistemic humility threaten religious beliefs?. J. Psychol. Theol..

[bb0100] Dworsky C.K.O., Pargament K.I., Gibbel M.R., Krumrei E.J., Faigin C.A., Haugen M.R.G., Desai K.M., Lauricella S.K., Lynn Q., Warner H.L. (2013). Winding road: preliminary support for a spiritually integrated intervention addressing college students’ spiritual struggles. Res. Soc. Sci. Study Relig..

[bb0105] Ellison C.G., Fan D. (2008). Daily spiritual experiences and psychological well-being among US adults. Soc. Indic. Res..

[bb0110] Exline J.J., Pargament K.I., Grubbs J.B., Yali A.M. (2014). The Religious and Spiritual Struggles Scale: development and initial validation. Psychol. Relig. Spiritual..

[bb0115] Gómez J.M., Hendy N.T., Montargot N. (2023). Developing participant intellectual humility through technology delivered instruction – a proposed model. Int. J. Manag. Educ..

[bb0120] Grossmann I., Dorfman A., Oakes H., Santos H.C., Vohs K.D., Scholer A.A. (2021). Training for wisdom: the distanced-self-reflection diary method. Psychol. Sci..

[bb0125] Haggard M., Rowatt W.C., Leman J.C., Meagher B., Moore C., Fergus T., Whitcomb D., Battaly H., Baehr J., Howard-Snyder D. (2018). Finding middle ground between intellectual arrogance and intellectual servility: development and assessment of the limitations-owning intellectual humility scale. Personal. Individ. Differ..

[bb0130] Hall M.E. (2021). Teaching students to doubt well: the roles of intellectual humility and uncertainty tolerance. Christ. Scholar’s Rev..

[bb0135] Hanel P.H.P., Roy D., Taylor S., Franjieh M., Heffer C., Tanesini A., Maio G.R. (2023). Using self-affirmation to increase intellectual humility in debate. R. Soc. Open Sci..

[bb0140] Haney A.M., Rollock D. (2020). A matter of faith: the role of religion, doubt, and personality in emerging adult mental health. Psychol. Relig. Spiritual..

[bb0145] Harris J.I., Usset T., Voecks C., Thuras P., Currier J., Erbes C. (2018). Spiritually integrated care for PTSD: a randomized controlled trial of “Building Spiritual Strength.”. Psychiatry Res..

[bb0150] Hook J.N., Davis D.E., Van Tongeren D.R., Hill P.C., Worthington E.L., Farrell J.E., Dieke P. (2015). Intellectual humility and forgiveness of religious leaders. J. Posit. Psychol..

[bb0155] Huynh A.C., Yang D.Y.-J., Grossmann I. (2016). The value of prospective reasoning for close relationships. Soc. Psychol. Personal. Sci..

[bb0160] Ji Q., Janicke-Bowles S.H., De Leeuw R.N.H., Oliver M.B. (2019). The melody to inspiration: the effects of awe-eliciting music on approach motivation and positive well-being. Media Psychol..

[bb0165] Kim Y., Nusbaum H.C., Yang F. (2022). Going beyond ourselves: the role of self-transcendent experiences in wisdom. Cognit. Emot..

[bb0170] Kim E., Salcone S., Fernandez P.E., Currier J.M. (2025). Psychological Trauma: Theory, Research, Practice, and Policy.

[bb0175] Krumrei-Mancuso E.J. (2017). Intellectual humility and prosocial values: direct and mediated effects. J. Posit. Psychol..

[bb0180] Krumrei-Mancuso E.J., Rouse S.V. (2016). The development and validation of the Comprehensive Intellectual Humility Scale. J. Pers. Assess..

[bb0190] Krumrei-Mancuso E.J., Haggard M.C., LaBouff J.P., Rowatt W.C. (2020). Links between intellectual humility and acquiring knowledge. J. Posit. Psychol..

[bb0195] Krumrei-Mancuso E.J., Trammell J., Harriger J.A. (2023). Affective, cognitive, and environmental inductions of humility and intellectual humility that center on self-transcendence. J. Posit. Psychol..

[bb0200] Krumrei-Mancuso E.J., Pärnamets P., Bland S., Astola M., Cichocka A., de Ridder J., Mercier H., Meyer M., O’Connor C., Porter T., Tanesini A., Alfano M., Van Bavel J.J. (2025). Toward an understanding of collective intellectual humility. Trends Cogn. Sci..

[bb0205] Krumrei-Mancuso E.J., Trammell J.P., Harriger J.A., Evans J.A. (2025). Replicating and extending research on sanctification: a cognitive appraisal with implications for behaviors, attitudes, and self-image. Int. J. Clin. Health Psychol..

[bb0210] Krumrei-Mancuso E.J., Trammell J.P., Harriger J.A., Evans J.A. (2025). A qualitative examination of sanctification: sources and varieties of appraisals of sacredness. Int. J. Clin. Health Psychol..

[bb0185] Krumrei-Mancuso E.J., Worthington E.L., Ottati V., Stern C. (2023). Divided: Open-mindedness and Dogmatism in a Polarized World.

[bb0215] Leary M.R., Diebels K.J., Davisson E.K., Jongman-Sereno K.P., Isherwood J.C., Raimi K.T., Deffler S.A., Hoyle R.H. (2017). Cognitive and interpersonal features of intellectual humility. Personal. Soc. Psychol. Bull..

[bb0220] Lehmann M., Genzer S., Kassem N., Van Tongeren D.R., Perry A. (2025). Intellectual humility predicts empathic accuracy and empathic resilience. Personal. Soc. Psychol. Bull..

[bb0225] Levi R., Ridberg R., Akers M., Seligman H. (2021). Survey fraud and the integrity of web-based survey research. Am. J. Health Promot..

[bb0230] Löchner J., Carlbring P., Björn S., Torous J., Sander L.B. (2025). Digital interventions in mental health: an overview and future perspectives. Internet Interv..

[bb0235] Lockhart K.L., Goddu M.K., Smith E.D., Keil F.C. (2016). What could you really learn on your own?: understanding the epistemic limitations of knowledge acquisition. Child Dev..

[bb0240] Mak W.W., Cheung R.Y. (2010). Self-stigma among concealable minorities in Hong Kong: conceptualization and unified measurement. Am. J. Orthop..

[bb0245] Marie L.G., Perez J.I., Guedes de Menezes I., Judd C.D., Beltran S.J. (2022). Evidence that intellectual humility can be heightened via a self-affirmation induction. Psi Beta J. Stud. Res..

[bb0250] Martino A.S., Perrotta A., McGillion B.J. (2024). Who can you trust these days?: dealing with imposter participants during online recruitment and data collection. Qual. Res..

[bb0255] Meagher B.R., Leman J.C., Bias J.P., Latendresse S.J., Rowatt W.C. (2015). Contrasting self-report and consensus ratings of intellectual humility and arrogance. J. Res. Pers..

[bb0260] Meagher B.R., Leman J.C., Heidenga C.A., Ringquist M.R., Rowatt W.C. (2020). Intellectual humility in conversation: distinct behavioral indicators of self and peer ratings. J. Posit. Psychol..

[bb0265] Mendonça S.E., Murray Dykhuis E., Jayawickreme E. (2023). Examining the possibilities for volitional character change in compassion and intellectual humility through a three-month online intervention. J. Res. Pers..

[bb0270] Milner K., Crawford P., Edgley A., Hare-Duke L., Slade M. (2019). The experiences of spirituality among adults with mental health difficulties: a qualitative systematic review. Epidemiol. Psychiatr. Sci..

[bb0275] Moreno R., Mayer R.E. (1999). Cognitive principles of multimedia learning: the role of modality and contiguity. J. Educ. Psychol..

[bb0280] Murdoch-Gibson S. (2022). Here for the incentive: recognizing and rooting out fake respondents. https://www.qrcaviews.org/2022/03/02/here-for-the-incentive-recognizing-and-rooting-out-fake-respondents/.

[bb0285] Murray-Swank N.A., Pargament K.I. (2005). God, where are you?: evaluating a spiritually-integrated intervention for sexual abuse. Ment. Health Relig. Cult..

[bb0290] Ochieng C.A., Minion J.T., Turner A., Blell M., Murtagh M.J. (2021). What does engagement mean to participants in longitudinal cohort studies? A qualitative study. BMC Med. Ethics.

[bb0295] Oxhandler H.K., Parrish D.E., Torres L.R., Achenbaum W.A. (2015). The integration of clients’ religion and spirituality in social work practice: a national survey. Soc. Work.

[bb0300] Pargament K.I. (1997).

[bb0305] Pargament K.I. (2007).

[bb0310] Pargament K.I., Exline J.J., Moreira-Almeida A., Mosqueiro B.P., Bhurgra D. (2021). Spirituality and Mental Health Across Cultures.

[bb0315] Pargament K.I., Oman D., Pomerleau J., Mahoney A. (2017). Some contributions of a psychological approach to the study of the sacred. Religion.

[bb0320] Pollet T.V., Saxton T.K. (2019). How diverse are the samples used in the journals ‘*Evolution & Human Behavior*’ and ‘*Evolutionary Psychology*’?. Evol. Psychol. Sci..

[bb0325] Porter T., Schumann K. (2017). Intellectual humility and openness to the opposing view. Self Identity.

[bb0330] Porter T., Baldwin C.R., Warren M.T., Murray E.D., Cotton Bronk K., Forgeard M.J.C., Snow N.E., Jayawickreme E. (2021). Clarifying the content of intellectual humility: a systematic review and integrative framework. J. Pers. Assess..

[bb0335] Reist G.M., Regueiro V., Pargament K.I. (2019). A spiritually integrated intervention for spiritual struggles among adults with mental illness: results of an initial evaluation. Spiritual. Clin. Pract..

[bb0340] Rodriguez D., Hook J.N., Farrell J.E., Mosher D.K., Zhang H., Van Tongeren D.R., Hill P.C. (2017). Religious intellectual humility, attitude change, and closeness following religious disagreement. J. Posit. Psychol..

[bb0345] Saleem M., Kühne L., De Santis K.K., Christianson L., Brand T., Busse H. (2021). Understanding engagement strategies in digital interventions for mental health promotion: scoping review. JMIR Mental Health.

[bb0350] Saulsman L.M. (2025). Using metaphor to facilitate cognitive detachment in cognitive behaviour therapies. Cogn. Behav. Ther..

[bb0355] Schafer R.M., Handal P.J., Brawer P.A., Ubinger M. (2011). Training and education in religion/spirituality within APA-accredited clinical psychology programs: 8 years later. J. Relig. Health.

[bb0360] Schmeichel B.J., Vohs K. (2009). Self-affirmation and self-control: affirming core values counteracts ego depletion. J. Pers. Soc. Psychol..

[bb0365] Sedlar A.E., Stauner N., Pargament K.I., Exline J.J., Grubbs J.B., Bradley D.F. (2018). Spiritual struggles among atheists: links to psychological distress and well-being. Religions.

[bb0370] Skitka L.J., Hanson B.E., Morgan G.S., Wisneski D.C. (2021). The psychology of moral conviction. Annu. Rev. Psychol..

[bb0375] Stauner N., Exline J.J., Wilt J.A., Vail K.E., Routledge C. (2020). The Science of Religion, Spirituality, and Existentialism.

[bb0380] Stott R., Mansell W., Salkovskis P., Lavender A., Cartwright-Hatton S. (2010).

[bb0385] Teitcher J.E., Bockting W.O., Bauermeister J.A., Hoefer C.J., Miner M.H., Klitzman R.L. (2015). Detecting, preventing, and responding to “fraudsters” in internet research: ethics and tradeoffs. J. Law Med. Ethics.

[bb0390] Upadhyay U.D., Lipkovich H. (2020). Using online technologies to improve diversity and inclusion in cognitive interviews with young people. BMC Med. Res. Methodol..

[bb0395] Van Tongeren D.R., Green J.D., Hulsey T.L., Legare C.H., Bromley D.G., Houtman A.M. (2014). A meaning-based approach to humility: relationship affirmation reduces worldview defense. J. Psychol. Theol..

[bb0400] VanderWeele T.J. (2017). Religious communities and human flourishing. Curr. Dir. Psychol. Sci..

[bb0405] Vieten C., Scammell S., Pierce A., Pilato R., Ammondson I., Pargament K.I., Lukoff D. (2016). Competencies for psychologists in the domains of religion and spirituality. Spiritual. Clin. Pract..

[bb0410] Welker K.M., Duong M., Rakhshani A., Dieffenbach M., Coleman P., Haidt J. (2023). The online educational program ‘perspectives’ improves affective polarization, intellectual humility, and conflict management. J. Soc. Polit. Psychol..

[bb0415] Wilt J.A., Exline J.J., Pargament K.I. (2022). Daily measures of religious/spiritual struggles: relations to depression, anxiety, satisfaction with life, and meaning. Psychol. Relig. Spiritual..

[bb0420] Zhang H., Farrell J.E., Hook J.N., Davis D.E., Van Tongeren D.R., Johnson K.A. (2015). Intellectual humility and forgiveness of religious conflict. J. Psychol. Theol..

